# (3*R*,3a*R*,6*R*,6a*R*)-Hexa­hydro­furo[3,2-*b*]furan-3,6-diyl dibenzoate

**DOI:** 10.1107/S1600536813021612

**Published:** 2013-08-07

**Authors:** Vincenzo Piccialli, Sabrina Zaccaria, Nicola Borbone, Roberto Centore, Angela Tuzi

**Affiliations:** a Dipartimento di Scienze Chimiche, Università degli Studi di Napoli ’Federico II’, Complesso di Monte S. Angelo, Via Cinthia, 80126 Napoli, Italy; b Dipartimento di Farmacia, Università degli Studi di Napoli ’Federico II’, Via D. Montesano 49, 80131 Napoli, Italy

## Abstract

The title compound, C_20_H_18_O_6_, prepared from d-mannitol by a two-step procedure, is a functionalized fused bis-tetra­hydro­furan. In the central fragment, consisting of two fused tetra­hydro­furan rings, one O atom and its two adjacent C atoms, a methyl­ene and a bridgehead C atom, are disordered over two sets of sites with an occupancy ratio of 0.735 (9):0.265 (9). In the major component, the ring containing the disordered O atom is a half-chair conformation with twisted methylene and benzoate-substituted C atoms, whereas the other ring has a half-chair or T-form conformation. In the minor component, the ring with the disordered O atom has an envelope conformation, with the O atom as the flap, and the other ring has a half-chair conformation, with the O atom and the other bridgehead CH atom being twisted. The two aromatic rings are inclined to one another by 20.00 (12)°. In the crystal, adjacent molecules are linked *via* C—H⋯π interactions, forming chains propagating along [010].

## Related literature

For the use of carbohydrates in the synthesis of complex natural chiral substances, see: Hanessian (1993 ▶). For mannitol as a chiral reagent and for its biologically active derivatives, see: Babjak *et al.* (2002 ▶); Masaki *et al.* (1999 ▶); Lohray *et al.* (1999 ▶). For oxidative processes mediated by transition of oxo-species, see: Piccialli, Oliviero *et al.* (2013 ▶); Piccialli, Tuzi *et al.* (2013 ▶); Piccialli, D’Errico *et al.* (2013 ▶); Piccialli *et al.* (2012 ▶). For the synthesis of the title compound, see: Hockett *et al.* (1946 ▶).
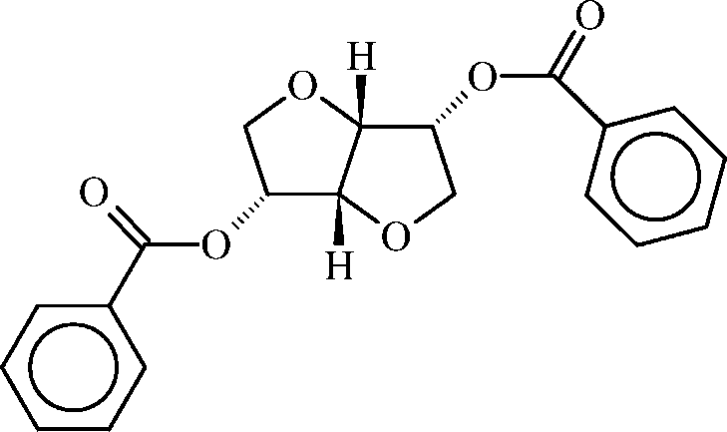


## Experimental

### 

#### Crystal data

C_20_H_18_O_6_
*M* *_r_* = 354.34
Monoclinic, 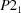

*a* = 10.0914 (15) Å
*b* = 8.2388 (11) Å
*c* = 10.7592 (10) Å
β = 108.913 (10)°
*V* = 846.24 (19) Å^3^
*Z* = 2
Mo *K*α radiation
μ = 0.10 mm^−1^
*T* = 173 K
0.50 × 0.20 × 0.10 mm

#### Data collection

Bruker–Nonius KappaCCD diffractometer
Absorption correction: multi-scan (*SADABS*; Bruker, 2001 ▶) *T*_min_ = 0.950, *T*_max_ = 0.990
7903 measured reflections
2059 independent reflections
1757 reflections with *I* > 2σ(*I*)
*R*_int_ = 0.033

#### Refinement

*R*[*F* ^2^ > 2σ(*F* ^2^)] = 0.035
*wR*(*F* ^2^) = 0.073
*S* = 1.11
2059 reflections
263 parameters
12 restraints
H-atom parameters constrained
Δρ_max_ = 0.14 e Å^−3^
Δρ_min_ = −0.20 e Å^−3^

### 

Data collection: *COLLECT* (Nonius, 1999 ▶); cell refinement: *DIRAX*/*LSQ* (Duisenberg *et al.*, 2000 ▶); data reduction: *EVALCCD* (Duisenberg *et al.*, 2003 ▶); program(s) used to solve structure: *SIR97* (Altomare *et al.*, 1999 ▶); program(s) used to refine structure: *SHELXL97* (Sheldrick, 2008 ▶); molecular graphics: *ORTEP-3 for Windows* (Farrugia, 2012 ▶); software used to prepare material for publication: *WinGX* (Farrugia, 2012 ▶).

## Supplementary Material

Crystal structure: contains datablock(s) global, I. DOI: 10.1107/S1600536813021612/yk2098sup1.cif

Structure factors: contains datablock(s) I. DOI: 10.1107/S1600536813021612/yk2098Isup2.hkl

Supplementary material file. DOI: 10.1107/S1600536813021612/yk2098Isup3.cml

Additional supplementary materials:  crystallographic information; 3D view; checkCIF report

## Figures and Tables

**Table 1:** Hydrogen-bond geometry (Å, °) *Cg* is the centroid of the C15–C20 ring.

*D*—H⋯*A*	*D*—H	H⋯*A*	*D*⋯*A*	*D*—H⋯*A*
C1—H1*A*⋯*Cg* ^i^	0.99	2.60	3.419 (3)	149

Symmetry code: (i) 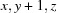.

**Fig. 1.**
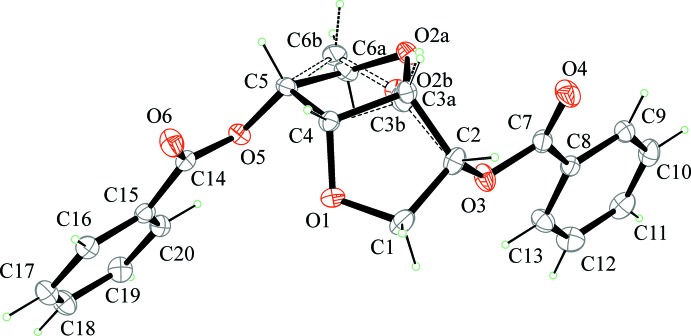
ORTEP view of the title compound, with the atom labelling scheme. Thermal ellipsoids are drawn at 30% probability level. The disordered part with the minor occupancy factor is drawn by dashed lines.

## supplementary crystallographic information

### 1. Comment

Carbohydrate-based synthons have been used in a number of syntheses of complex natural chiral substances (Hanessian, 1993). Manipulation of the oxygenation pattern and stereochemistry of simple sugars proved to be a powerful mean to use nature-generated chirality. In this context D-mannitol plays an important role as a readily available chiral building block in organic synthesis (Babjak *et al.*, 2002). In addition, mannitol and its derivatives are widely used as chiral reagents and chiral auxiliaries (Masaki *et al.*, 1999) and can be transformed into biologically active and pharmaceutically important compounds (Lohray *et al.*, 1999). As a continuation of our interest in oxidative processes mediated by transition metals oxo-species (Piccialli, Oliviero, Borbone *et al.*, 2013; Piccialli, Tuzi, Oliviero *et al.*, 2013; Piccialli, D'Errico, Borbone *et al.*, 2013; Piccialli *et al.*, 2012) we were interested in the synthesis and reactivity of functionalized fused bis-tetrahydrofurans.

In the title compound, molecule consists of two *cis*–fused tetrahydrofurane rings substituted at C2 and C5 positions (Fig.1). A local C_2_ symmetry of the molecule is observed at the junction. Tetrahydrofurane rings are disordered in two different positions (occupancy factor refined to 0.735 (9) for the A position and 0.265 (9) for the B position). Both disordered O1/C1/C2/C3A/C4 and O1/C1/C2/C3B/C4 rings adopt an envelope conformation with O1 at the flap. The disordered O2A/C3A/C4/C5/C6A and O2B/C3B/C4/C5/C6B rings are both in the envelope conformation that differ for the atom at the flap (C6A and O2B, respectively). The molecule of the title compound does not contain strong H–bonding donor and the crystal packing is stabilized by normal weak intermolecular interactions.

### 2. Experimental

The title compound was prepared according to a two-steps procedure starting from D-mannitol. Treatment of the latter with benzoyl chloride in pyridine for 16 h, followed by reaction with catalytic amounts of *p*-toluenesulfonic acid in 1,1,2,2-tetrachloroethane at reflux (Hockett *et al.*, 1946) led to title compound as an amorphous white powder, after chromatography (CHCl_3_ to CHCl_3_–MeOH, 95:5). The whole process consists of a double migration of the primary benzoates to the adjacent alcoholic functions followed by double water elimination and bis-cyclization (C1/C4 and C3/C6 ether ring closure). Recrystallization from methanol gave elongated white crystals suitable for X-ray analysis.

1,4:3,6-dianhydro-2,5-di-*O*-benzoyl-D-mannitol: ^1^H-NMR (200 MHz, CDCl_3_) δ 8.10 (4*H*, d, *J* = 7.0 Hz), 7.58 (2*H*, t, *J* = 7.3 Hz), 7.45 (4*H*, t, *J* = 7.3 Hz), 5.42–5.27 (m, 2H), 4.95–4.83 (m, 2H), 4.15 (2*H*, dd, *J* = 9.4, 6.3 Hz), 4.02 (2*H*, dd, *J* = 9.4, 6.7 Hz); ^13^C-NMR (50 MHz, CDCl_3_) δ 165.9, 133.2, 129.8, 129.4, 128.4, 80.6, 74.1, 70.7.

### 3. Refinement

All H atoms were generated stereochemically and refined by the riding model with *U*_iso_=1.2×*U*_eq_ of the carrier atom. Some constraints were introduced in the last stage of refinement to handle the disorder of one furane group (SAME and ISOR command of *SHELXL* program). In the absence of strong anomalous scatterer the Flack parameter is not meaningful. Data were merged using MERG 3 instruction and the absolute configuration was assigned on the assumption that the original mannitol configuration had been preserved during the acid-catalysed process.

### Crystal data

**Table:**

C_20_H_18_O_6_	*F*(000) = 372
*M**_r_* = 354.34	*D*_x_ = 1.391 Mg m^−^^3^
Monoclinic, *P*2_1_	Mo *K*α radiation, λ = 0.71073 Å
Hall symbol: P 2yb	Cell parameters from 446 reflections
*a* = 10.0914 (15) Å	θ = 3.8–23.5°
*b* = 8.2388 (11) Å	µ = 0.10 mm^−^^1^
*c* = 10.7592 (10) Å	*T* = 173 K
β = 108.913 (10)°	Block, white
*V* = 846.24 (19) Å^3^	0.50 × 0.20 × 0.10 mm
*Z* = 2	

### Data collection

**Table:**

Bruker–Nonius KappaCCD diffractometer	2059 independent reflections
Radiation source: normal-focus sealed tube	1757 reflections with *I* > 2σ(*I*)
Graphite monochromator	*R*_int_ = 0.033
Detector resolution: 9 pixels mm^-1^	θ_max_ = 27.5°, θ_min_ = 3.2°
CCD rotation images, thick slices scans	*h* = −12→13
Absorption correction: multi-scan (*SADABS*; Bruker, 2001)	*k* = −10→9
*T*_min_ = 0.950, *T*_max_ = 0.990	*l* = −13→13
7903 measured reflections	

### Refinement

**Table:**

Refinement on *F*^2^	Primary atom site location: structure-invariant direct methods
Least-squares matrix: full	Secondary atom site location: difference Fourier map
*R*[*F*^2^ > 2σ(*F*^2^)] = 0.035	Hydrogen site location: inferred from neighbouring sites
*wR*(*F*^2^) = 0.073	H-atom parameters constrained
*S* = 1.11	*w* = 1/[σ^2^(*F*_o_^2^) + (0.0287*P*)^2^ + 0.1292*P*] where *P* = (*F*_o_^2^ + 2*F*_c_^2^)/3
2059 reflections	(Δ/σ)_max_ = 0.001
263 parameters	Δρ_max_ = 0.14 e Å^−^^3^
12 restraints	Δρ_min_ = −0.20 e Å^−^^3^

### Special details

**Table:**

Geometry. All s.u.'s (except the s.u. in the dihedral angle between two l.s. planes) are estimated using the full covariance matrix. The cell s.u.'s are taken into account individually in the estimation of s.u.'s in distances, angles and torsion angles; correlations between s.u.'s in cell parameters are only used when they are defined by crystal symmetry. An approximate (isotropic) treatment of cell s.u.'s is used for estimating s.u.'s involving l.s. planes.
Refinement. Refinement of *F*^2^ against ALL reflections. The weighted *R*-factor *wR* and goodness of fit *S* are based on *F*^2^, conventional *R*-factors *R* are based on *F*, with *F* set to zero for negative *F*^2^. The threshold expression of *F*^2^ > σ(*F*^2^) is used only for calculating *R*-factors(gt) *etc*. and is not relevant to the choice of reflections for refinement. *R*-factors based on *F*^2^ are statistically about twice as large as those based on *F*, and *R*- factors based on ALL data will be even larger.

### Fractional atomic coordinates and isotropic or equivalent isotropic displacement parameters (Å^2^)

**Table:**

	*x*	*y*	*z*	*U*_iso_*/*U*_eq_	Occ. (<1)
O1	0.64835 (16)	0.0655 (2)	0.86046 (14)	0.0337 (4)	
O3	0.77544 (15)	0.3383 (2)	0.73544 (16)	0.0351 (4)	
O4	0.68459 (17)	0.4767 (2)	0.54748 (17)	0.0449 (5)	
O5	0.69994 (15)	−0.22410 (18)	0.78559 (13)	0.0282 (3)	
O6	0.51687 (15)	−0.2810 (2)	0.85248 (15)	0.0385 (4)	
C1	0.6475 (3)	0.2374 (3)	0.8702 (2)	0.0381 (6)	
H1A	0.7348	0.2762	0.9366	0.046*	
H1B	0.5668	0.2741	0.8961	0.046*	
C2	0.6365 (2)	0.3022 (3)	0.7353 (2)	0.0322 (5)	
H2	0.5777	0.4027	0.7162	0.039*	
C4	0.5552 (2)	0.0247 (3)	0.73299 (19)	0.0252 (4)	
H4	0.4576	0.0078	0.7343	0.030*	
C5	0.6094 (2)	−0.1245 (3)	0.68230 (19)	0.0267 (5)	
H5	0.5316	−0.1890	0.6207	0.032*	
C7	0.7845 (2)	0.4329 (3)	0.6365 (2)	0.0305 (5)	
C8	0.9319 (2)	0.4765 (3)	0.6514 (2)	0.0278 (4)	
C9	0.9560 (2)	0.5661 (3)	0.5518 (2)	0.0339 (5)	
H9	0.8800	0.5972	0.4769	0.041*	
C10	1.0912 (3)	0.6098 (3)	0.5623 (2)	0.0422 (6)	
H10	1.1082	0.6704	0.4938	0.051*	
C11	1.2014 (3)	0.5664 (3)	0.6708 (3)	0.0436 (6)	
H11	1.2941	0.5973	0.6774	0.052*	
C12	1.1774 (2)	0.4781 (4)	0.7702 (3)	0.0459 (6)	
H12	1.2537	0.4490	0.8456	0.055*	
C13	1.0429 (2)	0.4318 (3)	0.7606 (2)	0.0362 (6)	
H13	1.0266	0.3696	0.8286	0.043*	
C14	0.6400 (2)	−0.2914 (3)	0.8676 (2)	0.0277 (5)	
C15	0.7421 (2)	−0.3791 (3)	0.97809 (19)	0.0271 (5)	
C16	0.6933 (2)	−0.4500 (3)	1.0721 (2)	0.0301 (5)	
H16	0.5982	−0.4367	1.0668	0.036*	
C17	0.7827 (2)	−0.5397 (3)	1.1732 (2)	0.0355 (5)	
H17	0.7489	−0.5880	1.2373	0.043*	
C18	0.9210 (3)	−0.5593 (3)	1.1812 (2)	0.0391 (6)	
H18	0.9822	−0.6218	1.2503	0.047*	
C19	0.9706 (2)	−0.4876 (3)	1.0884 (2)	0.0381 (6)	
H19	1.0660	−0.5003	1.0945	0.046*	
C20	0.8815 (2)	−0.3974 (3)	0.9868 (2)	0.0302 (5)	
H20	0.9157	−0.3483	0.9234	0.036*	
C3A	0.5619 (7)	0.1641 (7)	0.6404 (6)	0.0262 (11)	0.735 (9)
H3A	0.4650	0.1986	0.5873	0.031*	0.735 (9)
C6A	0.7006 (7)	−0.0439 (7)	0.6118 (7)	0.0287 (14)	0.735 (9)
H6A	0.7949	−0.0213	0.6745	0.034*	0.735 (9)
H6B	0.7108	−0.1161	0.5419	0.034*	0.735 (9)
O2A	0.6338 (4)	0.1033 (3)	0.5563 (3)	0.0297 (9)	0.735 (9)
C3B	0.5953 (15)	0.150 (2)	0.650 (2)	0.040 (6)	0.265 (9)
H3B	0.5162	0.1724	0.5676	0.048*	0.265 (9)
C6B	0.687 (2)	−0.0796 (14)	0.587 (2)	0.039 (6)	0.265 (9)
H6C	0.7746	−0.1422	0.6041	0.047*	0.265 (9)
H6D	0.6276	−0.0933	0.4946	0.047*	0.265 (9)
O2B	0.7130 (10)	0.0860 (10)	0.6220 (9)	0.032 (3)	0.265 (9)

### Atomic displacement parameters (Å^2^)

**Table:**

	*U*^11^	*U*^22^	*U*^33^	*U*^12^	*U*^13^	*U*^23^
O1	0.0432 (9)	0.0322 (9)	0.0240 (7)	−0.0038 (7)	0.0085 (6)	−0.0056 (7)
O3	0.0256 (8)	0.0375 (9)	0.0432 (9)	−0.0012 (7)	0.0124 (7)	0.0140 (8)
O4	0.0315 (9)	0.0482 (11)	0.0457 (9)	−0.0019 (8)	−0.0005 (7)	0.0154 (9)
O5	0.0292 (7)	0.0308 (8)	0.0247 (7)	0.0038 (6)	0.0089 (6)	0.0030 (6)
O6	0.0261 (8)	0.0441 (10)	0.0437 (9)	−0.0019 (8)	0.0090 (7)	0.0120 (8)
C1	0.0452 (14)	0.0340 (14)	0.0420 (13)	−0.0139 (11)	0.0237 (11)	−0.0139 (11)
C2	0.0268 (11)	0.0274 (12)	0.0467 (13)	−0.0037 (9)	0.0177 (10)	−0.0038 (11)
C4	0.0222 (10)	0.0274 (11)	0.0261 (10)	−0.0028 (8)	0.0080 (8)	−0.0003 (9)
C5	0.0280 (11)	0.0297 (12)	0.0200 (9)	0.0010 (9)	0.0045 (8)	0.0010 (9)
C7	0.0305 (11)	0.0249 (12)	0.0346 (11)	−0.0017 (9)	0.0087 (9)	0.0018 (10)
C8	0.0292 (10)	0.0259 (11)	0.0286 (10)	−0.0026 (9)	0.0097 (8)	−0.0005 (9)
C9	0.0425 (13)	0.0308 (13)	0.0302 (11)	−0.0025 (11)	0.0140 (10)	0.0022 (10)
C10	0.0555 (15)	0.0351 (14)	0.0491 (14)	−0.0068 (12)	0.0349 (13)	0.0006 (12)
C11	0.0352 (13)	0.0444 (15)	0.0590 (15)	−0.0105 (12)	0.0260 (12)	−0.0118 (14)
C12	0.0294 (12)	0.0591 (18)	0.0469 (14)	−0.0013 (13)	0.0090 (10)	−0.0015 (14)
C13	0.0303 (11)	0.0452 (15)	0.0331 (11)	−0.0011 (11)	0.0103 (9)	0.0079 (11)
C14	0.0308 (11)	0.0235 (11)	0.0280 (10)	−0.0030 (9)	0.0087 (9)	−0.0030 (9)
C15	0.0299 (10)	0.0233 (11)	0.0270 (10)	−0.0002 (9)	0.0076 (8)	−0.0037 (9)
C16	0.0302 (11)	0.0302 (12)	0.0317 (11)	0.0038 (10)	0.0125 (9)	−0.0014 (10)
C17	0.0407 (13)	0.0367 (13)	0.0325 (11)	0.0058 (11)	0.0164 (10)	0.0058 (11)
C18	0.0381 (13)	0.0402 (15)	0.0364 (12)	0.0090 (11)	0.0084 (10)	0.0104 (11)
C19	0.0287 (12)	0.0403 (14)	0.0446 (13)	0.0046 (10)	0.0108 (10)	0.0031 (12)
C20	0.0300 (11)	0.0297 (12)	0.0314 (10)	−0.0010 (9)	0.0107 (9)	0.0004 (10)
C3A	0.027 (2)	0.025 (2)	0.028 (2)	0.0013 (19)	0.012 (2)	0.0032 (17)
C6A	0.034 (2)	0.034 (3)	0.021 (3)	0.009 (2)	0.0130 (18)	0.000 (2)
O2A	0.0313 (19)	0.0332 (13)	0.0277 (15)	0.0016 (10)	0.0137 (15)	0.0044 (10)
C3B	0.041 (7)	0.036 (7)	0.041 (7)	0.006 (4)	0.010 (5)	0.002 (4)
C6B	0.068 (11)	0.029 (7)	0.024 (8)	0.006 (6)	0.020 (7)	−0.009 (6)
O2B	0.026 (5)	0.040 (5)	0.033 (5)	0.001 (3)	0.014 (4)	−0.001 (3)

### Geometric parameters (Å, º)

**Table:**

O1—C1	1.421 (3)	C11—C12	1.378 (4)
O1—C4	1.429 (2)	C11—H11	0.9500
O3—C7	1.345 (3)	C12—C13	1.381 (3)
O3—C2	1.433 (2)	C12—H12	0.9500
O4—C7	1.199 (3)	C13—H13	0.9500
O5—C14	1.341 (3)	C14—C15	1.485 (3)
O5—C5	1.444 (2)	C15—C20	1.387 (3)
O6—C14	1.203 (3)	C15—C16	1.389 (3)
C1—C2	1.516 (3)	C16—C17	1.381 (3)
C1—H1A	0.9900	C16—H16	0.9500
C1—H1B	0.9900	C17—C18	1.379 (3)
C2—C3B	1.530 (13)	C17—H17	0.9500
C2—C3A	1.551 (5)	C18—C19	1.386 (3)
C2—H2	1.0000	C18—H18	0.9500
C4—C3B	1.50 (2)	C19—C20	1.384 (3)
C4—C5	1.516 (3)	C19—H19	0.9500
C4—C3A	1.536 (7)	C20—H20	0.9500
C4—H4	1.0000	C3A—O2A	1.422 (5)
C5—C6B	1.520 (6)	C3A—H3A	1.0000
C5—C6A	1.522 (4)	C6A—O2A	1.421 (6)
C5—H5	1.0000	C6A—H6A	0.9900
C7—C8	1.489 (3)	C6A—H6B	0.9900
C8—C13	1.385 (3)	C3B—O2B	1.418 (9)
C8—C9	1.386 (3)	C3B—H3B	1.0000
C9—C10	1.380 (3)	C6B—O2B	1.416 (10)
C9—H9	0.9500	C6B—H6C	0.9900
C10—C11	1.373 (4)	C6B—H6D	0.9900
C10—H10	0.9500		
			
C1—O1—C4	106.71 (18)	C11—C12—H12	119.9
C7—O3—C2	115.89 (17)	C13—C12—H12	119.9
C14—O5—C5	115.68 (16)	C12—C13—C8	119.7 (2)
O1—C1—C2	106.30 (19)	C12—C13—H13	120.2
O1—C1—H1A	110.5	C8—C13—H13	120.2
C2—C1—H1A	110.5	O6—C14—O5	123.3 (2)
O1—C1—H1B	110.5	O6—C14—C15	124.1 (2)
C2—C1—H1B	110.5	O5—C14—C15	112.60 (17)
H1A—C1—H1B	108.7	C20—C15—C16	119.72 (19)
O3—C2—C1	107.69 (19)	C20—C15—C14	122.18 (19)
O3—C2—C3B	104.2 (5)	C16—C15—C14	118.04 (18)
C1—C2—C3B	101.9 (10)	C17—C16—C15	120.2 (2)
O3—C2—C3A	114.8 (3)	C17—C16—H16	119.9
C1—C2—C3A	104.0 (3)	C15—C16—H16	119.9
O3—C2—H2	110.1	C18—C17—C16	120.0 (2)
C1—C2—H2	110.1	C18—C17—H17	120.0
C3B—C2—H2	121.9	C16—C17—H17	120.0
C3A—C2—H2	110.1	C17—C18—C19	120.0 (2)
O1—C4—C3B	100.9 (7)	C17—C18—H18	120.0
O1—C4—C5	109.53 (17)	C19—C18—H18	120.0
C3B—C4—C5	98.3 (4)	C20—C19—C18	120.2 (2)
O1—C4—C3A	107.1 (3)	C20—C19—H19	119.9
C5—C4—C3A	106.0 (2)	C18—C19—H19	119.9
O1—C4—H4	111.3	C19—C20—C15	119.8 (2)
C3B—C4—H4	124.1	C19—C20—H20	120.1
C5—C4—H4	111.3	C15—C20—H20	120.1
C3A—C4—H4	111.3	O2A—C3A—C4	106.7 (4)
O5—C5—C4	113.31 (16)	O2A—C3A—C2	116.0 (4)
O5—C5—C6B	108.7 (9)	C4—C3A—C2	103.6 (4)
C4—C5—C6B	111.6 (5)	O2A—C3A—H3A	110.1
O5—C5—C6A	107.3 (3)	C4—C3A—H3A	110.1
C4—C5—C6A	99.9 (3)	C2—C3A—H3A	110.1
O5—C5—H5	111.9	O2A—C6A—C5	107.5 (3)
C4—C5—H5	111.9	O2A—C6A—H6A	110.2
C6B—C5—H5	98.5	C5—C6A—H6A	110.2
C6A—C5—H5	111.9	O2A—C6A—H6B	110.2
O4—C7—O3	123.5 (2)	C5—C6A—H6B	110.2
O4—C7—C8	124.4 (2)	H6A—C6A—H6B	108.5
O3—C7—C8	112.14 (18)	C6A—O2A—C3A	107.7 (4)
C13—C8—C9	120.1 (2)	O2B—C3B—C4	106.1 (13)
C13—C8—C7	122.15 (19)	O2B—C3B—C2	110.4 (9)
C9—C8—C7	117.73 (19)	C4—C3B—C2	106.3 (13)
C10—C9—C8	119.5 (2)	O2B—C3B—H3B	111.3
C10—C9—H9	120.3	C4—C3B—H3B	111.3
C8—C9—H9	120.3	C2—C3B—H3B	111.3
C11—C10—C9	120.6 (2)	O2B—C6B—C5	98.6 (6)
C11—C10—H10	119.7	O2B—C6B—H6C	112.0
C9—C10—H10	119.7	C5—C6B—H6C	112.0
C10—C11—C12	120.0 (2)	O2B—C6B—H6D	112.0
C10—C11—H11	120.0	C5—C6B—H6D	112.0
C12—C11—H11	120.0	H6C—C6B—H6D	109.7
C11—C12—C13	120.2 (2)	C6B—O2B—C3B	108.5 (8)
			
C4—O1—C1—C2	−36.6 (2)	C14—C15—C16—C17	176.8 (2)
C7—O3—C2—C1	−164.35 (19)	C15—C16—C17—C18	0.0 (4)
C7—O3—C2—C3B	87.9 (10)	C16—C17—C18—C19	0.6 (4)
C7—O3—C2—C3A	80.4 (4)	C17—C18—C19—C20	−0.6 (4)
O1—C1—C2—O3	−95.8 (2)	C18—C19—C20—C15	−0.1 (4)
O1—C1—C2—C3B	13.5 (5)	C16—C15—C20—C19	0.7 (3)
O1—C1—C2—C3A	26.3 (3)	C14—C15—C20—C19	−176.7 (2)
C1—O1—C4—C3B	43.2 (4)	O1—C4—C3A—O2A	108.9 (3)
C1—O1—C4—C5	146.16 (18)	C3B—C4—C3A—O2A	46 (3)
C1—O1—C4—C3A	31.6 (3)	C5—C4—C3A—O2A	−8.0 (4)
C14—O5—C5—C4	63.8 (2)	O1—C4—C3A—C2	−14.1 (4)
C14—O5—C5—C6B	−171.5 (7)	C3B—C4—C3A—C2	−77 (3)
C14—O5—C5—C6A	173.1 (3)	C5—C4—C3A—C2	−130.9 (3)
O1—C4—C5—O5	23.7 (2)	O3—C2—C3A—O2A	−6.4 (6)
C3B—C4—C5—O5	128.4 (7)	C1—C2—C3A—O2A	−123.8 (4)
C3A—C4—C5—O5	138.9 (3)	C3B—C2—C3A—O2A	−42 (5)
O1—C4—C5—C6B	−99.4 (11)	O3—C2—C3A—C4	110.2 (3)
C3B—C4—C5—C6B	5.3 (13)	C1—C2—C3A—C4	−7.2 (4)
C3A—C4—C5—C6B	15.8 (11)	C3B—C2—C3A—C4	75 (5)
O1—C4—C5—C6A	−90.2 (3)	O5—C5—C6A—O2A	−153.4 (4)
C3B—C4—C5—C6A	14.6 (8)	C4—C5—C6A—O2A	−35.0 (6)
C3A—C4—C5—C6A	25.0 (4)	C6B—C5—C6A—O2A	109 (5)
C2—O3—C7—O4	−5.1 (3)	C5—C6A—O2A—C3A	32.0 (7)
C2—O3—C7—C8	174.68 (19)	C4—C3A—O2A—C6A	−14.5 (6)
O4—C7—C8—C13	175.5 (2)	C2—C3A—O2A—C6A	100.3 (6)
O3—C7—C8—C13	−4.2 (3)	O1—C4—C3B—O2B	84.2 (10)
O4—C7—C8—C9	−3.9 (3)	C5—C4—C3B—O2B	−27.7 (11)
O3—C7—C8—C9	176.4 (2)	C3A—C4—C3B—O2B	−156 (4)
C13—C8—C9—C10	0.2 (4)	O1—C4—C3B—C2	−33.4 (9)
C7—C8—C9—C10	179.6 (2)	C5—C4—C3B—C2	−145.2 (7)
C8—C9—C10—C11	−0.6 (4)	C3A—C4—C3B—C2	87 (3)
C9—C10—C11—C12	0.2 (4)	O3—C2—C3B—O2B	9.7 (18)
C10—C11—C12—C13	0.5 (4)	C1—C2—C3B—O2B	−102.2 (15)
C11—C12—C13—C8	−0.9 (4)	C3A—C2—C3B—O2B	157 (7)
C9—C8—C13—C12	0.5 (4)	O3—C2—C3B—C4	124.4 (6)
C7—C8—C13—C12	−178.9 (2)	C1—C2—C3B—C4	12.4 (9)
C5—O5—C14—O6	5.3 (3)	C3A—C2—C3B—C4	−88 (5)
C5—O5—C14—C15	−174.67 (17)	O5—C5—C6B—O2B	−107.4 (12)
O6—C14—C15—C20	175.5 (2)	C4—C5—C6B—O2B	18.3 (17)
O5—C14—C15—C20	−4.5 (3)	C6A—C5—C6B—O2B	−20 (3)
O6—C14—C15—C16	−1.9 (3)	C5—C6B—O2B—C3B	−37 (2)
O5—C14—C15—C16	178.10 (19)	C4—C3B—O2B—C6B	44.0 (17)
C20—C15—C16—C17	−0.6 (3)	C2—C3B—O2B—C6B	158.7 (14)

### Hydrogen-bond geometry (Å, º)

Cg is the centroid of the C15–C20 ring.

**Table:**

*D*—H···*A*	*D*—H	H···*A*	*D*···*A*	*D*—H···*A*
C1—H1*A*···*Cg*^i^	0.99	2.60	3.419 (3)	149

Symmetry code: (i) *x*, *y*+1, *z*.
